# PREDICTED DIFFERENCES OF EPIGENETIC AGE AND DEMENTIA RISK AMONG DIFFERENT TRAJECTORIES OF LONGITUDINAL LONELINESS

**DOI:** 10.1093/geroni/igac059.2079

**Published:** 2022-12-20

**Authors:** Morgan Lynch, Christopher Beam

**Affiliations:** University of Southern California, Los Angeles, California, United States; University of Southern California, Los Angeles, California, United States

## Abstract

Different trajectories of loneliness in late adulthood may explain differences in the effects of aging on dementia risk. We tested whether greater or increasing loneliness across three time points demonstrated stronger associations between aging and dementia risk in a sample of 1,814 Health and Retirement Study participants. Dementia risk was quantified using modified Telephone Interview for Cognitive Status scores (TICSm), age was quantified using the epigenetic clock DNAm PhenoAge, and loneliness was measured with the UCLA Loneliness short-form scale. Growth mixture modeling was used to identify loneliness latent classes that best represented trajectories according to model fit statistics. Five groups were identified: low loneliness, high declining, low increasing, moderate and stable, and a moderate declining group. Multivariate analysis of variance was used to test whether class membership differentially predicted TICSm scores, PhenoAge, and the correlation between TICSm and PhenoAge.TICSm scores were statistically significantly lower (worse) for the high-declining compared to the low (-1.08 95% CI [-1.75, -0.42]) and low-increasing groups (-1.63 95% CI [-2.28, -0.98]), and the moderate group was lower than the low-increasing group (-1.11 95% CI [-1.66, -0.57] and the low group (-0.57 [-1.13, -0.002]) (p’s < 0.05). No significant differences in PhenoAge or the correlation between PhenoAge and TICSm were found between groups. Analyses statistically adjusted for demographic characteristics, objective social isolation, depression, BMI, smoking, self-rated health, and polygenic risk score for cognition. Results suggest that epigenome-wide ages are unlikely to mediate the relationship between loneliness and dementia risk.

